# The public health response to an outbreak of border-spill malaria along China-Myanmar border

**DOI:** 10.1371/journal.pone.0275932

**Published:** 2022-12-16

**Authors:** Zu-Rui Lin, Shan-Shan Yin, Jie Yang, Xiang-Rui Guo, Chao-Liang Dong, Ying-Kun Lin, Chun-Li Ding, Xiao-Dong Sun, Run-Xian Yan, Suo-Lan Yang, Xian-Hua Zhou, Jian-Wei Xu

**Affiliations:** 1 Malaria Division, Yunnan Institute of Parasitic Diseases, Yunnan Provincial Centre of Malaria Research, Yunnan Provincial Key Laboratory of Vector-borne Disease Control and Research, Yunnan Institute of Parasitic Diseases Innovative Team of Key Techniques for Vector Borne Disease Control and Prevention, Training Base of International Scientific Exchange and Education in Tropical Diseases for South and Southeast Asia, Pu’er, Yunnan, China; 2 Parasitic Disease Section, Yingjiang County Center for Disease Control and Prevention, Yingjiang, Yunnan, China; 3 Parasitic Disease Section, Donghong Prefecture Center for Disease Control and Prevention, Mangshi, Yunnan, China; Fundação Oswaldo Cruz Centro de Pesquisas René Rachou: Fundacao Oswaldo Cruz Instituto Rene Rachou, BRAZIL

## Abstract

**Introduction:**

Malaria importation can be caused by cross-border movement either of both people and anopheline mosquitoes. However, there still lacks robust evidence of imported malaria caused by *Plasmodium* spp. infected *anopheles* along international border areas (border-spill malaria). The objectives of this study were to confirm whether an outbreak of *Plasmodium vivax* malaria is border-spill malaria and assess the effects of China’s public health response along China-Myanmar border.

**Methods:**

Epidemiological, parasitological and entomological investigations were conducted to investigate the outbreak of border-spill malaria. Meanwhile, comprehensive interventions were carried out to prevent further transmission and reintroduction of malaria.

**Results:**

Rapid diagnostic testing, microscopy and polymerase chain reaction were performed and the infections were confirmed as *P*. *vivax*. A total of 22 (9.21%) of 239 workers contracted *P*. *vivax* during the outbreak. Multivariate logistic regression analysis identified that the distance of worker shelters in China within 300 meters to the internally displaced person (IDP) camps in Myanmar was a risk factors associated with malaria infection (adjusted odds ratio 7.5920; 95% confidence interval, 2.6079–22.1013; *P* = 0.0002). After comprehensive interventions, malaria transmission was successfully interpreted and prevented at the project site till the completion of project on 14 January 2020, and recurrence of *P*. *vivax* malaria was not detected by the end of 2020.

**Conclusion:**

This study provided robust evidence of border-spill malaria along China-Myanmar border. Malaria parasite reservoir and distance travelled by female anopheline mosquitoes are two determinants for border-spill malaria. The public health response to the outbreak indicates that the malaria surveillance and response system works well in preventing reintroduction of malaria. However, prevention of border-spill malaria is still a major challenge in the Yunnan border area, China.

## Introduction

Malaria continues to be one of the leading public health threats in the world [1]. China has been reported malaria free since 2017 [2] and the World Health Organization (WHO) announced China as a malaria-free country in June, 2021 [3]. However, Yunnan Province of China shares 4060 kilometers of border with Myanmar, Laos and Vietnam. The hot climate, adequate precipitation and lush forests provide a suitable environment for the growth and reproduction of mosquitoes and for malaria transmission in Yunnan border area. With a complex vector community, *Anopheles minimus* and *An*. *sinensis* were identified as the primary and secondary vectors of malaria in the border area [4]. *An*. *kunmingensis*, *An*. *anthropophagus*, *An*. *dirus*, *An*. *jeyporiensis*, *An*. *pseudowillmori* and *An*. *maculatus* were also identified playing roles in malaria transmission [5]. Malaria importation can be caused by cross-border movements of humans and anopheline mosquitoes. The WHO also mentioned that malaria can spill over international borders from the border areas of endemic countries to neighboring countries [6]. This phenomenon has been known as border-spill malaria. Xu et al. defined border-spill malaria as a type of imported malaria carried by infected anophelines which fly over the boundary from endemic areas of neighboring countries to reintroduce malaria along the international border areas [7].

Cases about imported malaria caused by human migrants are broadly reported [4, 5, 7–16]. These cases are mainly found in Southeast Asia and South America. However, robust evidence has still been lacking on border-spill malaria. In early November 2019, the workers of a river embankment construction project were working on the boundary riverbank between China and Myanmar. The project site was 17 kilometers away from the nearest Chinese residential site, but there were two groups of internally displaced people (IDP) living in resettlement sites along the border riverbank in Myanmar. An outbreak of *Plasmodium vivax* malaria occurred among workers of the river embankment construction in November, 2019. The outbreak provided a representative example of the border-spill malaria. Strict border restrictions, under the context of the COVID-19 pandemic, dramatically reduced cross-border movement of human populations, and border-spill malaria becomes one of the major threats in preventing reintroduction of malaria transmission in the border area [4, 5, 7]. The objectives of this study were to carry out an investigation of causes of border-spill malaria and China’s public health response to the outbreaks.

## Methods

### Outbreak site

**Fig 1:**
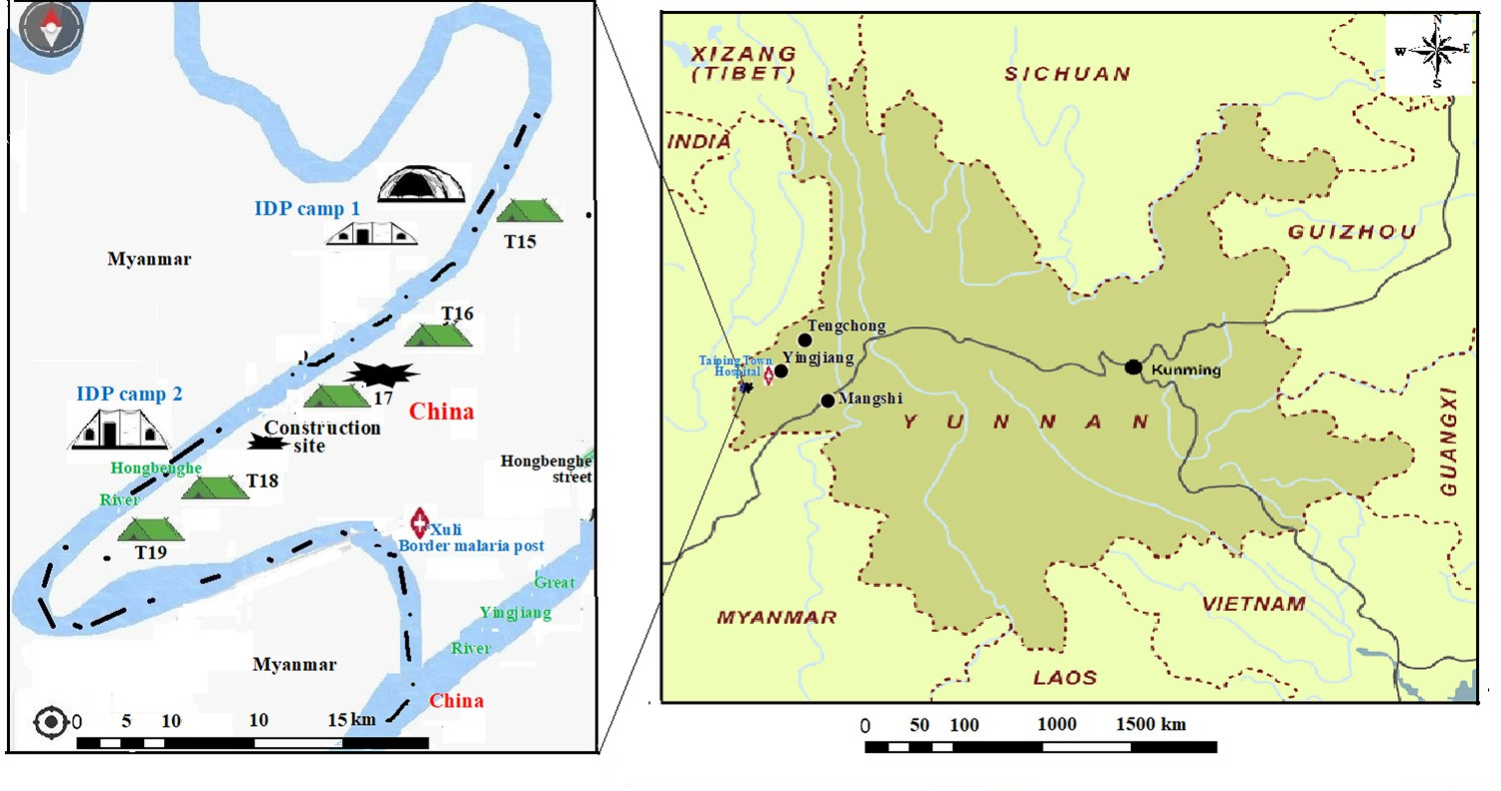
The geographical location of the project site and nearby areas, and the locations of internally displaced person (IDP) camps and worker team (T) shelters.

The outbreak of border-spill malaria was reported at the project site of Hongbenghe Water Conservancy Construction. The project site was about 17 kilometers away from the nearest residential site (Hongbenghe Street) and about 70 kilometers away from the closest infectious disease case registry site (Taiping Town hospital) in China (Fig 1). The workers were recruited from areas of Myanmar near the project site, and three counties of China, namely, Mangshi (which is about 250 kilometers away from the project site), Tengchong (which is about 170 kilometers away from the project site) and Yingjiang (which is about 80 kilometers away from the project site) (Fig 1). The project site was in a river valley with an altitude of 320–380 meters and subtropical rainforest climate. There are not any communities or residential sites near the section of the border river on China’s side. However, there are two IDP camps in Myanmar, along the border riverbank. The five kilometer long construction project was divided into five sections evenly. Five temporary worker teams (T15-T19) were recruited working on the river embankment construction project. One team is responsible for one section. The shelters of T15 and T18 were located at less than 300 meters away from IDP camp 1 and 2, respectively (Fig 1).

### Outbreak investigation

#### Focus identification

From 21 through 24 November 2019, eight malaria cases were reported to the China Information System for Disease Control and Prevention (CISDCP). They were detected by microscopy at Mangshi Center for Disease Control and Prevention (CDC), Yingjiang County Hospital, Tengchong Gudong Town Hospital and Yingjiang Xuli Border Malaria Post, respectively (Fig 1). The results of individual case epidemiological investigations indicated that all eight malaria patients had a history of staying at the project site. Mangshi CDC and Yingjiang CDC inferred that the eight patients might have contracted malaria at the project site. The first task of the public health response is to screen all workers for malaria. After inquiring to the Yingjiang County Bureau of Water Conservancy about information of the project, Yingjiang CDC screened workers at the project site and workers who had left the project site in their Yingjiang hometowns. Mangshi CDC and Tengchong CDC also screened workers who had left the project site in their hometowns (Table 1).

**Table 1:** The river embankment construction; and malaria outbreak, detection and public health response from 27 October, 2019 to 14 January, 2020.

Date	Activities
**27 Oct 2019**	A five kilometer long river embankment project rolled out in Yingjiang County, China. The project was divided into five sections. One worker team was enrolled working on one kilomter long section of the embankment.
**1–5 Nov 2019**	A total of 239 workers entered and stationed at the project site. 230 of them were from three counties (Mangshi, Tengchong and Yingjiang) of China, and nine of them from areas of Myanmar near of the project site.
**16–23 Nov 2019**	A worker started fever on 16 November, and then more workers fell ill with fever. 36 Chinese workers returned to their hometowns for treatment, including 26 to Mangshi, two to Tengchong and eight to Yingjiang.
**21 Nov 2019**	Manshi CDC detectd a worker with *Plasmodium vivax* infection by microscopy, and then reported it to the CISDCP.
**22 Nov 2019**	Manshi CDC detected and reported another three *P*. *vivax* malaria cases to the CISDCP.
**22 Nov 2019**	Mangshi CDC carried out individual epidemiological surveys to the four malaria cases. Based on results of the surveys, Mangshi CDC inferred that they were contracted malaria at the project site, and notified Yingjiang CDC.
**23 Nov 2019**	Yingjiang County Hospital detected and reported another two *P*. *vivax* malaria cases to the CISDCP. The two malaria case patients had ever stayed at the project site too.
**24 Nov 2019**	Tengchong Gudong Town Hospital and Yingjiang Xuli Border Malaria Post detected one *P*. *vivax* malaria case, respectively. The two malaria patients had ever stayed at the project site too.
**24–25 Nov 2019**	Yingjiang CDC screened all 203 workers for malaria at the project site. Nine workers were detected with *P*. *vivax* infection. Three ultraviolet mosquito traps were used to collect mosquitoes for one night. Two *Anopheles minimus* (principal vector) were captured. IRS with beta-cyfluthrin was carried out to all 20 shelters. A total of 300 LLINs and 324 mosquito repellent creams were distributed to workers. Nine workers with *P*. *vivax* were treated with 1200 mg Chloroquine for three days plus 180 mg primaquine for eight days. MDA with 2880mg DHA-PPQ for 3 days was administrated to 194 workers with testing negative for malaria presumptive treatment. Face to face health education on malaria was administrated to all workers.
**25–30 Nov 2019**	Yingjiang County Hospital detected and reported another five *P*. *vivax* malaria cases to the CISDCP.
**14 Jan, 2020**	The completion of the project, all workers left the project site for homes.

**Note:** Center for Disease control and Prevention (CDC); China Information System for Disease Control and Prevention (CISDCP); Rapid diagnostic tests (RDT); Indoor residual spraying (IRS); Long-lasting insecticidal nets (LLIN); Mass drug administration (MDA); Dihydroartemisinin-piperaquine phosphate (DHA-PPQ).

#### Parasitological investigation

An important step of the public health response was to investigate number of malaria infected workers in the outbreak and estimate size of the outbreak at first. All the 239 workers were tested by both rapid diagnostic tests (RDTs, Wondfo Pf/Pan, China) and microscopy for malaria by 30 November 2019. Using RDTs promptly detected workers with malaria infection and allowed the administration of treatment in care point sites. Using microscopy to confirm the results of RDTs is one of requirements in the technical guidelines of malaria elimination in China [17, 18]. Following the technical guidelines of laboratory quality control for malaria elimination, all positive samples and 10% of negative samples of RDTs and microscopy were sent to the Yunnan Provincial Reference Laboratory for Malaria (YRL), where polymerase chain reaction (PCR) was performed for reconfirming the results of RDTs and microscopy [17, 18].

#### Treatment

All the worker patients testing positive in RDTs were given treatment for malaria. The positive RDT showed Pan (non-*Plasmodium falciparum*) and the microscopy confirmed that they were *Plasmodium vivax*. Based on the antimalarial drug policy in China [19, 20], *P*. *vivax* malaria cases were treated with 1200 mg Chloroquine (CQ) for three days plus 180 primaquine (PQ) for eight days. Focus mass drug administration (MDA) was given to all negative RDT workers for presumptive treatment for three days. The regimen of MDA was 2880mg dihydroartemisinin-piperaquine phosphate (DHA-PPQ). Each DHA-PPQ tablet contains 40 mg base dihydroartemisinin and 320 mg piperaquine phosphate [19, 20]. During treatment, the patients were followed up by telephone call to learn patients’ adherence to the treatment courses, treatment efficacy and potential side-effects.

#### Entomological survey and vector control

Three ultraviolet mosquito traps (Kung Fu Xiaoshuai, Wuhan Jixing, China) powered by solar panels, were used to collect mosquitoes at the project site from 7:00 pm of 24th of November to 8: 00 am of next day, 2019. Weather conditions did not allow charging the solar panels for additional nights; and the mosquito traps could not thereby be used to further collect mosquitoes. Based on foundation information on local abundance and population dynamics of *Anopheles* mosquitoes [21], indoor residual spraying (IRS) with beta-cyfluthrin (Wuhan Kemike Biomedical Technology, China) was carried out to all 20 worker shelters. Long-lasting insecticidal nets (LLINs) and mosquito repellent creams were also distributed to every worker at the project site.

#### Further prevention, follow up and radical treatment

**Fig 2:**
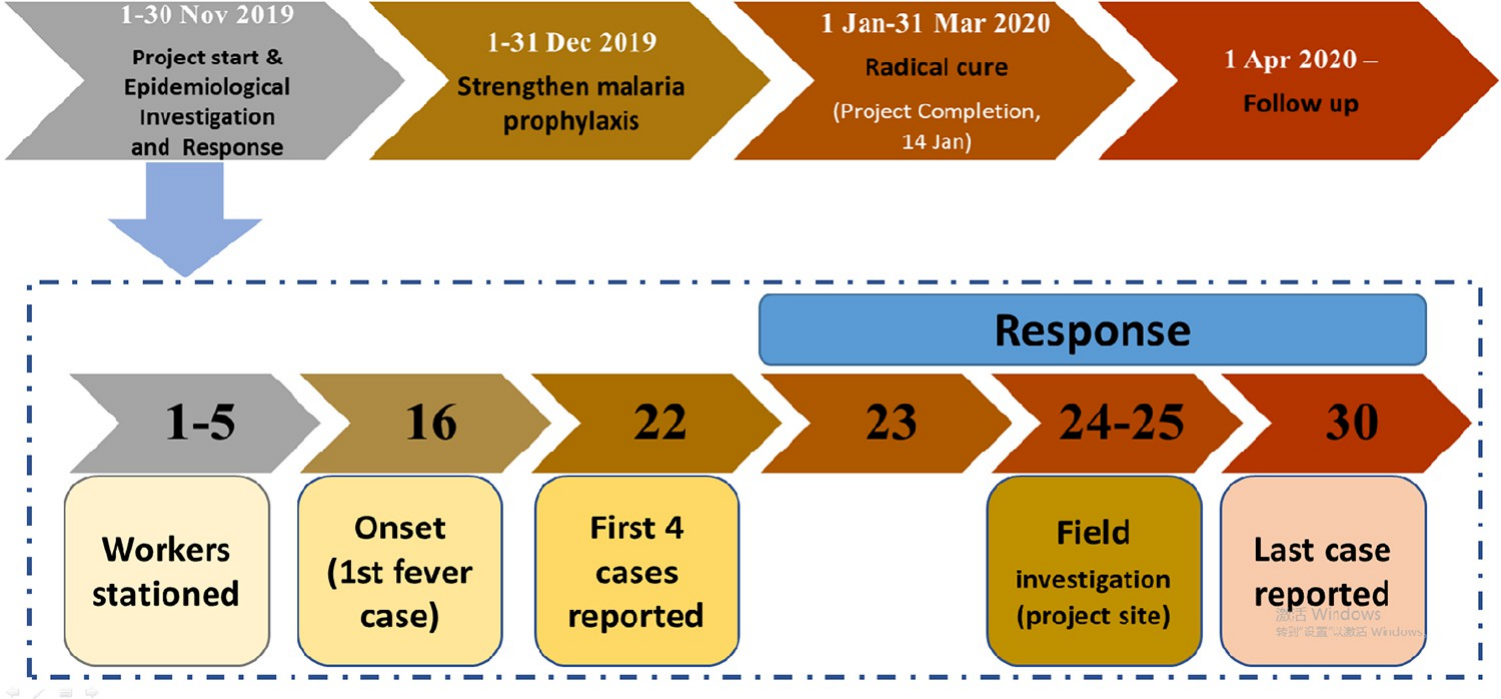
Process of the public health response to the border-spill malaria outbreak.

To promote individual malaria prevention, face-to-face health education on malaria, LLINs and mosquito repellent creams were provided to the workers at the project site. To prevent malaria transmission, a public health response was initiated to malaria cases detected in workers who were back in their homes according to the “1-3-7”working requirement from 23 to 30 November 2019 [7, 17]. The public health response included screening residents closely neighboring the homes of malaria cases and collecting anopheline mosquitoes at the malaria case locations in Mangshi, Tengchong and Yingjiang. RDTs were used for passive malaria case detection in the project site till the completion of the water conservancy construction project on 14 January 2019. Radical cure treatment with 180 mg PQ for eight days was administered to all the confirmed *P*. *vivax* malaria cases to prevent relapse in March 2020 [19, 20]. The Mangshi, Tengchong and Yingjiang county CDCs and township hospitals carried out active case detection to follow up the workers who ever stayed in the project site to the end of 2020 (Fig 2).

### Statistical analysis

The data were entered into Excel 2007, and statistical analysis was performed with Epi Info 7.2 (Centers for Disease Control and Prevention, USA). Slide positivity rates and their 95% confidence intervals (CI) were calculated. Chi-squared Fisher’s exact test was used to compare the proportions across sex, ages, nationalities, worker teams and distances to the IDP camps, respectively. A multivariate logistic regression analysis (MLA) was used to identify risk factors associated with malaria infections during the outbreak. In the MLA model, the outcome (dependent) variable was the result of microscopy, and independent variables were demographic characteristics, nationality, whether there were Burmese people in a worker team and the distance of a worker shelter in China to an IDP camp on the border river bank in Myanmar [22–24].

### Ethics

This study involved interventions for malaria elimination and thus ethical approval is not needed. The Ethics Committee of the Yunnan Institute of Parasitic Diseases (YIPD) waived the ethical approval for the public health response to the border-spill malaria outbreak prior to implementing the investigation. The protocol of outbreak investigation and public health response was consistent with the National Guidelines of Malaria Elimination in China. Based on the requirements of the Ethics Committee of the YIPD, the protocol was approved by Yingjiang County Bureau of Health. A verbal consent procedure for the outbreak survey and control interventions were also obtained from every worker and the community related prior to the implementation. All results were kept confidentially and would not be linked to any identifying individual information.

## Results

### Subject characteristics

The malaria focus was identified when Manshi CDC detected the first three *P*. *vivax* malaria cases and reported them to the CISDCP on 22 November. Among 239 workers who were temporarily enrolled in the river embankment construction project, nine (3.7%; 95%CI, 1.7–7.0%) were from areas of Myanmar near the project site, and 230 (96.2%; 95%CI, 93.0–98.3%) were from three counties of China, namely, Mangshi, Tengchong and Yingjiang. Most of them (87.92%; 95%CI, 83.0–91.7%) were men. The median age was 45 (rang, 16–64) years old. Most of them were farmers prior to working at the project site.

### Infection rate and risk factors

**Fig 3:**
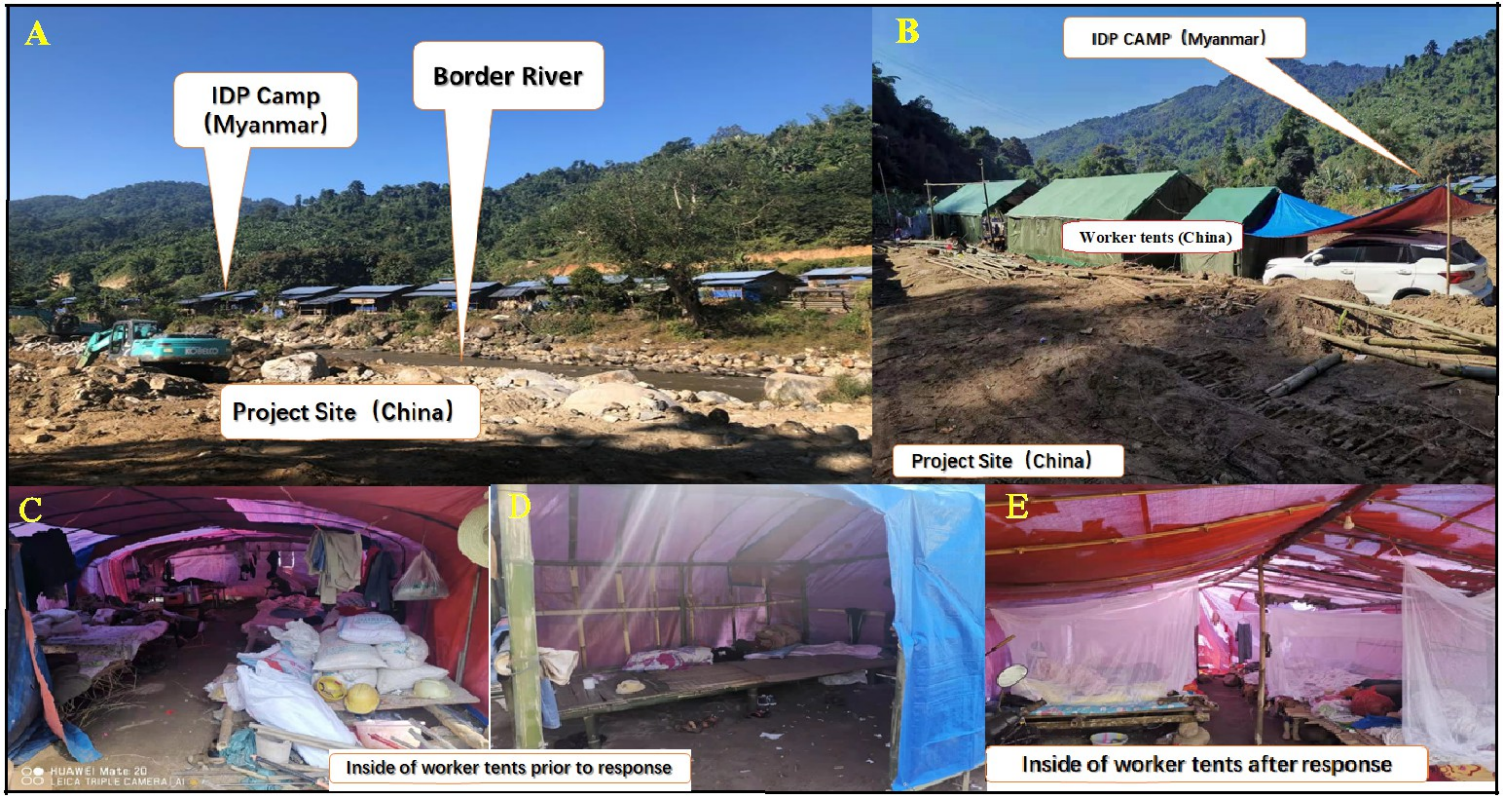
Pictures of worker’s residual conditions in the project sit. Internally Displaced Person (IDP); Worker Team (T).

All the 239 workers were screened for malaria, 203 tested at the project site and 36 tested in the health facilities of their hometowns. A total of 22 (9.21%; 95%CI, 5.86–13.60%) workers were detected with *P*. *vivax* infection, and they were confirmed by PCR at the YRL. The infection rates of malaria parasites were not significant considering sex, ages, nationality and some members of the worker team being Burmese (P> 0.05). However, the infection rates were significantly different among five worker teams (P < 0.01). The slide positivity rates of workers in T15 and T18 within a distance of 300 meters to an IDP camp, namely, 33.3% (95% CI, 17.29–52.81%) for T15 and 12.8% (95% CI, 4.8–25.7%) for T18, were higher than of other worker teams. Four (44.4%; 95% CI, 13.7–78.8%) of nine workers with Burmese nationality were detected with *P*. *vivax* infection (Table 2). The MLA produced adjusted odd ratio (AOR) 13.4962 (95% confidence interval [CI], 1.7640–103.2575; *P* = 0.0122) for workers with Burmese nationality, and AOR 7.5920 (95%CI, 2.6079–22.1013; *P* = 0.0002) for workers living in shelters within a distance of 300 meters to an IDP camp. These two factors were independently associated with malaria infection during the outbreak (Table 3). In the project site, all the workers were at a high risk of malaria infection owning to a lack of effective prevention actions. They lived in temporary shelters without bed nets (Fig 3). They did not adopt other prevention actions including use of chemoprophylaxis, mosquito coils and repellents.

**Table 2:** Results of microscopy for malaria parasites, including testing at the project site and health facilities at worker’s hometowns.

	No. tested	No. positivity	% (95%CI)	*P*-value
**Sex**				
**Female**	210	20	9.52 (5.91–14.33)	
**Male**	29	2	6.90 (0.85–22.77)	1.0000
**Age (years)**				
**16–30**	45	5	11.11 (3.71–24.05)	
**31–49**	108	14	12.96 (7.27–20.79)	
**≥50**	86	3	3.49 (0.73–9.86)	0.07344
**Nationality**				
**China**	230	18	7.83 (4.70–12.09)	
**Myanmar**	9	4	44.44 (13.70–78.80)	0.0051
**Work Team**				
**T15**	30	10	33.33 (17.29–52.81)	
**T16**	45	2	4.44 (0.54–15.15)	
**T17**	73	4	5.48 (1.51–13.44)	
**T18**	47	6	12.77 (4.83–25.74)	
**T19**	44	0	0 (0–8.04)	0.0009
**Worker teams with Burmese**				
**Yes**	72	9	12.50 (5.88–22.41)	
**No**	167	13	7.78 (4.21–12.94)	0.3284
**Distance to an internally displaced person camp on the river bank in Myanmar**				
**≤300m**	77	16	20.78 (12.37–31.54)	
**>300m**	162	6	3.70 (1.37–7.89)	0.0005

Note: 95% confidence interval (CI); Worker teams (T).

**Table 3:** Results of multivariate logistic regression analysis for risk factors associated with malaria infection during the border-malaria outbreak (N = 239).

	OR (95%CI)	*P*-value	AOR (95%CI)	*P*-value
**Sex**				
**Female**	1		1	
**Male**	1.4202(0.3143–6.4164)	0.6484	5.7292 (0.5759–56.9978)	0.1364
**Age (years)**				
**≥Age**	1		1	
**16–49**	3.9185(1.1255–13.6425)	0.0319	3.4354(0.9307–12.6801)	0.0640
**Nationality**				
**China**	1		1	
**Myanmar**	9.4278(2.3248–38.2326)	0.0017	13.4962(1.7640–103.2575)	0.0122
**Worker teams with Burmese**				
**No**	1		1	
**Yes**	1.6938(0.6895–4.1612)	0.2505	0.9100(0.2916–2.8401)	0.8710
**Distance to an internally displaced person camp on the riverbank in Myanmar**				
**>300m**	1		1	
**≤300m**	6.8181(2.5498–18.2314)	0.0001	7.5920 (2.6079–22.1013)	0.0002

Note: The outcome variable is microscopy results; Odds ratio (OR); Adjusted odds ratio (AOR); 95% confidence interval (CI).

### Intervention impacts

The epidemiological surveys of individual cases showed that all 22 *P*. *vivax* malaria cases had no history of malaria attack episodes prior to entering the project site. All the 18 Chinese malaria cases were cured with treatment regimen of 1200mg CQ for three days plus 180 mg PQ for eight days in November 2019, and followed by radical cure treatment with 180 mg PQ for eight days in March 2020. The four Burmese malaria cases were lost to follow up because they had been back to their homes in Myanmar. Thereby, the radical cure treatment with PQ could not be administered to the four malaria cases with Burmese nationality, and also there was no information to know whether they had been cured. Two *Anopheles minimus* mosquitoes (the local principal vector) were captured in the entomological survey. IRS with insecticide was thereby conducted to all the 20 shelters and a total of 300 LLINs (Fig 3) and 324 mosquito repellent creams were delivered to workers in the project site. Additionally, a face-to-face health education on malaria knowledge and individual protection against mosquito bites was administered to promote workers’ awareness and capacity in malaria prevention. No malaria case appeared at the project site in the period until completion of the river embankment construction project on 14 January 2020. In the six communities with malaria cases detected among the return workers, RDTs and microscopy were used to screen a total of 137 people including the return worker’s family members and their close neighbors for malaria. No malaria infections were detected. The MDA with 2880mg DHA-PPQ for presumptive treatment was administrated to 217 people with testing negative for malaria, including 194 workers at the project site and 23 family members of the return malaria cases in the six communities. The comprehensive interventions assured that no introduced and recurrent malaria cases were detected by the end of 2020.

## Discussion

### Border-spill malaria evidences

Transboundary malaria is a big challenge for malaria control and elimination [13–16], and it can be caused by people and anopheline mosquitoes [7]. Many literatures reported cross-border malaria infections through human movements along international borders in Southeast Asia [4, 5, 7–13] and Latin America [13–16]. The WHO mentioned that malaria can spill over international borders from the border areas of endemic countries into the border areas of neighboring countries through anopheline mosquitoes [6]. However, there still lacks evidence on mosquito-caused transboundary malaria, namely, the border-spill malaria. This study provided robust empirical evidence about the transboundary malaria caused by infected anopheline mosquitoes that crossed over the border from endemic areas of Myanmar to reintroduce malaria in the Yunnan border area, China. First, there were not any residential sites or communities within a 17-kilometer radius of the project site in China. Based on the results of 291 anopheline mosquito mark-release-recapture (MRR) experiments in 143 localities around the world, the estimated mean distance travelled by female anopheline mosquitoes can reach 2.5 km [25]. This can exclude the possibility of the *P*. *vivax* outbreak being the result of residual malaria parasites in Chinese populations in areas near the project site in China. Second, malaria transmission has been successfully interpreted and maintained malaria free from 17 April 2016 when the last case of indigenous malaria was reported in Yunnan [26]. Based on the criteria that local malaria transmission has been fully interrupted (zero indigenous human malaria cases) for at least the past 36 consecutive months, and an adequate program for preventing reintroduction of malaria transmission is fully functional throughout a county, the three counties (Mangshi, Tengchong and Yingjiang) where Chinese workers came from were locally certificated malaria free by China itself prior to October 2019 [7, 27–29]. Third, the two IDP camps were just stationed on the boundary riverbank in Myanmar. Kachin Special region II (KR2) is one of the main malaria hot spots [4, 5, 7] and continuous military conflict makes it difficult to effectively control malaria in the KR2 [29, 30]. Based on data from the cross-border joint prevention and control project of malaria and dengue fever in China (Yunnan)-GMS areas [31], the KR2 with a population of about 50,000 reported 2,080, 1,936 and 664 malaria cases in 2016, 2017 and 2018, respectively. Just prior to the border-spill malaria outbreak, a cross-sectional study detected a total of 200 *P*. *vivax* malaria cases in the IPD Camp 1 with a population of approximately 2,500 people in September 2019, and nine *P*. *vivax* malaria cases in the IPD Camp 2 with approximately 300 people in October 2019. The two IDP camps could be the malaria parasites reservoirs for the border-spill malaria outbreak. Fourth, all the 22 malaria case patients reported that they did not have any history of malaria attack episodes prior to entering the project site, the possibility of *P*. *vivax* recurrence or relapse could thereby be ruled out. Fifth, the first worker enterd the project site on 1^st^ November 2019. The first malaria patient began to have fever on 16 November 2019, and the last malaria case started fever on 26 November, an interval of only ten days. One of the biological characteristics of *P*. *vivax* is that the incubation period is more than 10 days, thus the interval between infection of people and appearance of clinical symptoms must be at least 10 days. Even in appropriate conditions of air temperature and relative humidity, sexual reproductive period of *P*. *vivax* in anopheline mosquitoes also needs at least 10 days. The reproduction of *P*. *vivax* malaria cases therefore needs more than 20 days [32, 33]. The biological characteristics of *P*. *vivax* do not support the possibility of any introduced malaria cases, namely, all the 22 *P*. *vivax* malaria cases were directly caused by *P*. *vivax* infected anopheline mosquitoes that flew over the boundary river. Sixth, although the investigation only captured two *An*. *minimus* mosquitoes in the project site, other studies demonstrated the abundance of vector anopheline mosquitoes in the region [21, 34]. Seventh, sixteen (72.7%) of the 22 cases were detected in T15 and T18 within 300 meters to the two IDP camps. The MLA also identified that the shelters within a distance of 300 meters to an IDP camp was one of risk factors associated with malaria infection during the outbreak. Last but not least, most of the workers might not have acquired functional immunity because they were enrolled from malaria free areas in China [32, 33]. They also lacked knowledge and awareness of malaria prevention. They stayed overnight in temporary shelters without using any chemoprevention, bed nets, mosquito coils or repellents.

When considering the border-spill malaria, the reservoir of malaria parasites and the travel distance of female anopheline mosquitoes are two determinant factors [11]. The border collaboration successfully reduced malaria burden along China-Myanmar border [7, 12]. However, the internal conflicts between the Kachin Independence Army and the Myanmar Government Defense Force [29] made malaria control interventions difficult and led to resurgence of malaria in the KR2 [30]. The abundance of malaria parasite reservoirs was one of key causes for the border-spill malaria outbreak. The worker shelters were within the normal 2.5 kilometer travel distance of the female anopheline mosquitoes [25]. The MLA results indicated that the workers in T15 and T18 within a distance of 300 meters to the two IDP camps were significantly at a higher risk of malaria infection. Ten (45.5%) of 22 malaria cases were detected among the workers in T15 neighboring with IDP camp 1, and six (27.3%) cases were detected among the workers in T18 neighboring with IDP camp 2. There were not any malaria cases detected from the workers in T19 with a distance of approximate 800 meters to IDP camp 2 (Fig 1, Table 2), which indicates the shorter distance to malaria parasite reservoirs, the higher risk of malaria infection. None of workers infected with malaria in T19 might be attributable to other factors. For example, the parasite amount and prevalence (3.0% [9/300]) in the IDP camp 2 was lower than that (8.0% [200/2500]) in the IDP camp 1. T19 shelters were located at the upper direction of wind and availability of a small forest between T19 shelters and IDP camp 2.

### Implication for the response to the border-spill malaria outbreak

This study provides an iconic example of border-spill malaria. The first worker entered the project site on 1^st^ November 2019, and then the first febrile patient appeared on 16 November 2019, but they were not the same people. The last malaria case started fever on 26 November and were detected with malaria on 30 November. The border-spill malaria outbreak lasted for 10 days and then was successfully contained. The successful public health response indicates that the malaria surveillance and response system work well in the Yunnan border area. It also documents that malaria hyperendemicity in some border parts of Myanmar is a continuous threat to reintroduction of malaria transmission in the border area, China. It also highlights the importance of continuous collaboration between neighboring countries for malaria control and elimination [7]. The multi-sector action has been regarded as one of main strategies in China, a total of 13 departments involved in malaria elimination programme [18]. However, the department of water conservancy is not one of 13 departments in the malaria elimination programme. Prior to the outbreak of border-spill malaria, the health sector did not have any information about the border river embankment project; and the department of water conservancy did not know that the workers needed prevention for malaria when they stationed on the border riverbank. Taking a lesson from the border-spill malaria outbreak, the health sector of Yingjiang County has established a link with the Yingjiang County Bureau of Water Conservancy. The higher level might be necessary to recognize the limitation in the multi-sector mechanism and update it for preventing reintroduction of malaria transmission.

In preventing reintroduction of malaria transmission, follow-up is one of the essential activities after a public health response to an outbreak. In this public health response, the treated patients were followed up by telephone call to learn patient adherence to the treatment courses, efficacy and side effect. To achieve timely detection of introduced malaria incidence resulting from imported malaria after the outbreak, the Mangshi, Tengchong and Yingjiang county CDCs and township hospitals carried out active case detections to follow up the workers who had ever stayed in the project site till the end of 2020. The follow-up strategy assured prevention of introduced malaria cases, and can be referred in other public health responses to infectious diseases (not just malaria).

### Challenges of border malaria prevention in context of the COVID-19 pandemic

The Yunnan border area is also one of the areas facing a high risk of the coronavirus disease 2019 (COVID-19) pandemic. To fight the COVID-19 pandemic, the border crossing regulations significantly reduced number of imported malaria cases carried by human border crossers. Border-spill malaria has become one of main threats in preventing reintroduction of malaria transmission in Yunnan [4, 5]. In 2021, Yingjiang County reported a total of 71 malaria cases, and 63 (88.7%) of them were categorized as border-spill malaria. The increased border-spill malaria cases were attributable to resurgent malaria in Myanmar KR2 neighboring with Yingjiang, China. Based on data exchange between Yunnan and the KR2 of Myanmar, the Laiza and nearby area in KR2 reported 250 malaria cases in 2019 followed by to 798 cases in 2020 to 1395 in 2021. Yunnan tried to communicate with the Health Authority of the KR2 to collaborate in rolling back the resurgent malaria, and suggested provision of drugs, insecticides, activity cost and technical assistance for malaria control in the KR2 in 2021. Due to health service disruptions and some resources moved from malaria control to the response to the COVID-19 pandemic, the Health Department of the Kachin Independent Organization responded that they were too busy responding to the COVID-19 pandemic to have human resources fighting malaria [4, 5]. Malaria incidence in the border areas of Myanmar near China is now continuously increasing. The 23 townships of Myanmar neighboring China reported 2,636 malaria cases in 2019, followed by to 4,046 cases in 2020 to 7,404 in 2021. In the context of the COVID-19 pandemic and border collaboration limitations for malaria, comprehensive intervention should be considered to prevent border-spill malaria in the Yunnan border area [4, 5, 7]. Meanwhile, China needs to be well prepared for fighting the threat of imported malaria in the post COVID-19 pandemic era [4, 5].

### Limitations

A wide variety of evidence, including entomology, epidemiology, parasitology and breeding sites of anopheline mosquitoes, are necessary to document border-spill malaria. This study provides sound evidence of border-spill malaria in parasitology and epidemiology along China-Myanmar border. However, this research still has limitations. A primary limitation of this study is that four (44.4%) of nine workers with Burmese nationality detected with *P*. *vivax* infection, but the investigation could not determine whether they were infected at the project site or their Myanmar hometowns. The first malaria case was not a worker with Burmese nationality and the outbreak occurred in such a short time following the worker entrance. Thus, the 18 malaria cases with Chinese nationality would not be introduced cases caused by the four malaria cases with Burmese nationality. The MLA also documented no association between the four malaria cases with Burmese nationality and others cases (AOR 0.9100; 95%CI, 0.2916–2.8401, *P* = 0.8710). The second limitation is the fact that the study investigated only five potential risk factors associated with malaria infection (sex, age, nationality, worker teams with Burmese people and distance to an IDP camp). However, all the workers stayed overnight in the temporary shelters without taking any preventive measures. Investigations on preventive measures may not be helpful for identifying risk factors associated with malaria infection in the outbreak. The third limitation is a lack of an appropriate entomological survey to collect enough data on anopheline mosquitoes in the investigation. Literatures reported that in Yunnan with a complex vector community, a total of eight species of anopheline mosquitoes were identified playing roles in malaria transmission [4, 5]. However, only two mosquitoes of *An*. *minimus* were captured in the public health response. Further entomological and ecological researches are necessary to provide solid evidence of border-spill malaria along the international border area. Trials of adult female anopheline mosquito MRR can provide ideal entomological evidence of the border-spill malaria. If adult female anopheline mosquitoes that marked and released in Myanmar border area were recaptured in the Yunnan border area, the results of MRR trials would provide the direct evidence that anopheline mosquitoes can cross the international border. If microscopy detected malaria parasite sporozoites in salivary glands of recaptured anopheline mosquitoes, and/or PCR test detected nucleic acids of parasite sporozoites and/or enzyme-linked immunosorbnent assay detected circumsporozoite proteins of parasite sporozoites in recaptured anopheline mosquitoes, the border-spill malaria would be affirmed in entomology. In this study, the mosquito traps were not able to use collecting mosquitoes due to a lack of electricity. However, anopheline mosquito breeding site surveys could be carried out to provide the vector larva data. Lacking the evidence of anopheline mosquito larvae could be one aspect of this limitation. More investigation needs to be done providing further evidence of border-spill malaria and its control. Adult female anopheline mosquitoes can fly as long as 2.5 kilometers [25]. To prevent the border-spill malaria and introduced cases, the proposed strategy is intensive interventions within 2.5 km-wide perimeter along the international border, including (1) proactive and passive malaria case detection, (2) intensive vector surveillance, (3) evidence-based vector control, and (4) evidence-based preventative treatment with anti-malarial drugs. Additionally, further solid collaboration between China and Myanmar is urgently needed for reduction of malaria burden, especially, rolling back the increasing malaria incidence trend, in the Myanmar border area [7].

## Conclusion

This study confirmed and characterized an outbreak of border-spill malaria, providing robust evidence of border-spill malaria along China-Myanmar border. Malaria parasite reservoir and the distance travelled by female anopheline mosquitoes are the two determinants for border-spill malaria. China’s public health response to the outbreak indicates that the malaria surveillance and response system work well in preventing reintroduction of malaria transmission in the Yunnan border area. Border-spill malaria is a big challenge which could reintroduce malaria transmission to the disease-free neighboring countries. Further solid collaboration between China and Myanmar and international support is needed to reduce malaria burden in the border areas of Myanmar.

## Supporting information

S1 Dataset (DOCX)
