# Supplementary material for: The public health response to an outbreak of border-spill malaria along China-Myanmar border
Source: PLoS One. 2022 Dec 16;17(12):e0275932. doi: 10.1371/journal.pone.0275932 (PMC9757579; doi:10.1371/journal.pone.0275932)
Supplement: S1 Dataset — (DOCX) [file pone.0275932.s001.docx]

**Dataset:**

**Table S1 Records of screening workers and interventions**

| **ID** | **Nationality (1=Chinese，2=Burmese)** | **RDT**  **(0=negative，1=** **positive)** | **Micros**  **(0=negative，1=** **positive)** | **Team number** | **Distance to IDP not more than 300 meter** | **Sex**  **（1 = f， 0 =m）** | **Project site Elevation（m）** | **Interventions（1=Yes, 0=No）** | | | | **Team With MMR people（1=Yes, 0=No）** |
| --- | --- | --- | --- | --- | --- | --- | --- | --- | --- | --- | --- | --- |
|  |  |  |  |  |  |  |  | **Preventive treatment** | **Repellent** | **Net** | **Other** |  |
| 1 | 1 |  | 1 | 15 | 1 | 1 | 364 | 1 | 1 | 1 | 1 | 1 |
| 2 | 1 | 0 | 0 | 15 | 1 | 1 | 364 | 1 | 1 | 1 | 1 | 1 |
| 3 | 1 | 0 | 0 | 15 | 1 | 1 | 364 | 1 | 1 | 1 | 1 | 1 |
| 4 | 1 |  | 1 | 15 | 1 | 1 | 364 | 1 | 1 | 1 | 1 | 1 |
| 5 | 1 |  | 1 | 15 | 1 | 1 | 364 | 1 | 1 | 1 | 1 | 1 |
| 6 | 1 |  | 1 | 15 | 1 | 1 | 364 | 1 | 1 | 1 | 1 | 1 |
| 7 | 1 | 0 | 0 | 15 | 1 | 1 | 364 | 1 | 1 | 1 | 1 | 1 |
| 8 | 1 | 0 | 0 | 15 | 1 | 1 | 364 | 1 | 1 | 1 | 1 | 1 |
| 9 | 1 | 0 | 0 | 15 | 1 | 1 | 364 | 1 | 1 | 1 | 1 | 1 |
| 10 | 1 | 0 | 0 | 15 | 1 | 1 | 364 | 1 | 1 | 1 | 1 | 1 |
| 11 | 1 | 0 | 0 | 15 | 1 | 1 | 364 | 1 | 1 | 1 | 1 | 1 |
| 12 | 1 | 0 | 0 | 15 | 1 | 1 | 364 | 1 | 1 | 1 | 1 | 1 |
| 13 | 1 | 0 | 0 | 15 | 1 | 1 | 364 | 1 | 1 | 1 | 1 | 1 |
| 14 | 1 | 0 | 0 | 15 | 1 | 1 | 364 | 1 | 1 | 1 | 1 | 1 |
| 15 | 1 | 0 | 0 | 15 | 1 | 1 | 364 | 1 | 1 | 1 | 1 | 1 |
| 16 | 1 | 0 | 0 | 15 | 1 | 1 | 364 | 1 | 1 | 1 | 1 | 1 |
| 17 | 1 | 0 | 0 | 15 | 1 | 1 | 364 | 1 | 1 | 1 | 1 | 1 |
| 18 | 1 | 0 | 0 | 15 | 1 | 1 | 364 | 1 | 1 | 1 | 1 | 1 |
| 19 | 1 | 0 | 0 | 15 | 1 | 1 | 364 | 1 | 1 | 1 | 1 | 1 |
| 20 | 1 | 0 | 0 | 15 | 1 | 1 | 364 | 1 | 1 | 1 | 1 | 1 |
| 21 | 1 | 0 | 0 | 15 | 1 | 2 | 364 | 1 | 1 | 1 | 1 | 1 |
| 22 | 2 |  | 1 | 15 | 1 | 2 | 364 | 1 | 1 | 1 | 1 | 1 |
| 23 | 2 |  | 1 | 15 | 1 | 2 | 364 | 1 | 1 | 1 | 1 | 1 |
| 24 | 1 |  | 1 | 15 | 1 | 1 | 364 | 1 | 1 | 1 | 1 | 1 |
| 25 | 1 | 0 | 0 | 16 | 0 | 1 | 364 | 1 | 1 | 1 | 1 | 1 |
| 26 | 1 | 0 | 0 | 16 | 0 | 1 | 364 | 1 | 1 | 1 | 1 | 1 |
| 27 | 1 | 0 | 0 | 16 | 0 | 1 | 364 | 1 | 1 | 1 | 1 | 1 |
| 28 | 2 |  | 1 | 16 | 0 | 1 | 364 | 1 | 1 | 1 | 1 | 1 |
| 29 | 2 |  | 1 | 16 | 0 | 1 | 364 | 1 | 1 | 1 | 1 | 1 |
| 30 | 1 | 0 | 0 | 16 | 0 | 1 | 364 | 1 | 1 | 1 | 1 | 1 |
| 31 | 1 | 0 | 0 | 16 | 0 | 1 | 364 | 1 | 1 | 1 | 1 | 1 |
| 32 | 1 | 0 | 0 | 16 | 0 | 1 | 364 | 1 | 1 | 1 | 1 | 1 |
| 33 | 1 | 0 | 0 | 16 | 0 | 1 | 364 | 1 | 1 | 1 | 1 | 1 |
| 34 | 1 | 0 | 0 | 16 | 0 | 1 | 364 | 1 | 1 | 1 | 1 | 1 |
| 35 | 1 | 0 | 0 | 16 | 0 | 1 | 364 | 1 | 1 | 1 | 1 | 1 |
| 36 | 1 | 0 | 0 | 16 | 0 | 1 | 364 | 1 | 1 | 1 | 1 | 1 |
| 37 | 1 | 0 | 0 | 16 | 0 | 1 | 364 | 1 | 1 | 1 | 1 | 1 |
| 38 | 1 | 0 | 0 | 16 | 0 | 2 | 364 | 1 | 1 | 1 | 1 | 1 |
| 39 | 1 | 0 | 0 | 16 | 0 | 1 | 364 | 1 | 1 | 1 | 1 | 1 |
| 40 | 1 | 0 | 0 | 16 | 0 | 1 | 364 | 1 | 1 | 1 | 1 | 1 |
| 41 | 1 | 0 | 0 | 16 | 0 | 1 | 364 | 1 | 1 | 1 | 1 | 1 |
| 42 | 1 | 0 | 0 | 16 | 0 | 1 | 364 | 1 | 1 | 1 | 1 | 1 |
| 43 | 1 | 0 | 0 | 16 | 0 | 1 | 364 | 1 | 1 | 1 | 1 | 1 |
| 44 | 1 | 0 | 0 | 16 | 0 | 1 | 364 | 1 | 1 | 1 | 1 | 1 |
| 45 | 1 | 0 | 0 | 16 | 0 | 1 | 364 | 1 | 1 | 1 | 1 | 1 |
| 46 | 1 | 0 | 0 | 16 | 0 | 2 | 364 | 1 | 1 | 1 | 1 | 1 |
| 47 | 1 | 0 | 0 | 16 | 0 | 1 | 364 | 1 | 1 | 1 | 1 | 1 |
| 48 | 1 | 0 | 0 | 16 | 0 | 1 | 364 | 1 | 1 | 1 | 1 | 1 |
| 49 | 1 | 0 | 0 | 16 | 0 | 1 | 364 | 1 | 1 | 1 | 1 | 1 |
| 50 | 1 | 0 | 0 | 16 | 0 | 1 | 364 | 1 | 1 | 1 | 1 | 1 |
| 51 | 1 | 0 | 0 | 16 | 0 | 1 | 364 | 1 | 1 | 1 | 1 | 1 |
| 52 | 1 | 0 | 0 | 16 | 0 | 1 | 364 | 1 | 1 | 1 | 1 | 1 |
| 53 | 1 | 0 | 0 | 16 | 0 | 2 | 364 | 1 | 1 | 1 | 1 | 1 |
| 54 | 1 | 0 | 0 | 16 | 0 | 1 | 364 | 1 | 1 | 1 | 1 | 1 |
| 55 | 1 | 0 | 0 | 16 | 0 | 2 | 364 | 1 | 1 | 1 | 1 | 1 |
| 56 | 1 | 0 | 0 | 16 | 0 | 1 | 364 | 1 | 1 | 1 | 1 | 1 |
| 57 | 1 | 0 | 0 | 16 | 0 | 1 | 364 | 1 | 1 | 1 | 1 | 1 |
| 58 | 1 | 0 | 0 | 17 | 0 | 1 | 364 | 1 | 1 | 1 | 1 |  |
| 59 | 1 | 0 | 0 | 17 | 0 | 1 | 364 | 1 | 1 | 1 | 1 |  |
| 60 | 1 | 0 | 0 | 17 | 0 | 1 | 364 | 1 | 1 | 1 | 1 |  |
| 61 | 1 | 0 | 0 | 17 | 0 | 2 | 364 | 1 | 1 | 1 | 1 |  |
| 62 | 1 | 0 | 0 | 17 | 0 | 1 | 364 | 1 | 1 | 1 | 1 |  |
| 63 | 1 | 0 | 0 | 17 | 0 | 1 | 364 | 1 | 1 | 1 | 1 |  |
| 64 | 1 | 0 | 0 | 17 | 0 | 1 | 364 | 1 | 1 | 1 | 1 |  |
| 65 | 1 | 0 | 0 | 17 | 0 | 1 | 364 | 1 | 1 | 1 | 1 |  |
| 66 | 1 | 0 | 0 | 17 | 0 | 1 | 364 | 1 | 1 | 1 | 1 |  |
| 67 | 1 | 0 | 0 | 17 | 0 | 1 | 364 | 1 | 1 | 1 | 1 |  |
| 68 | 1 | 0 | 0 | 17 | 0 | 1 | 364 | 1 | 1 | 1 | 1 |  |
| 69 | 1 | 0 | 0 | 17 | 0 | 1 | 364 | 1 | 1 | 1 | 1 |  |
| 70 | 1 | 0 | 0 | 17 | 0 | 1 | 364 | 1 | 1 | 1 | 1 |  |
| 71 | 1 | 0 | 0 | 17 | 0 | 1 | 364 | 1 | 1 | 1 | 1 |  |
| 72 | 1 | 0 | 0 | 17 | 0 | 1 | 364 | 1 | 1 | 1 | 1 |  |
| 73 | 1 | 0 | 0 | 17 | 0 | 1 | 364 | 1 | 1 | 1 | 1 |  |
| 74 | 1 | 0 | 0 | 17 | 0 | 1 | 364 | 1 | 1 | 1 | 1 |  |
| 75 | 1 | 0 | 0 | 17 | 0 | 1 | 364 | 1 | 1 | 1 | 1 |  |
| 76 | 1 | 0 | 0 | 17 | 0 | 2 | 364 | 1 | 1 | 1 | 1 |  |
| 77 | 1 | 0 | 0 | 17 | 0 | 1 | 364 | 1 | 1 | 1 | 1 |  |
| 78 | 1 | 0 | 0 | 17 | 0 | 1 | 364 | 1 | 1 | 1 | 1 |  |
| 79 | 1 | 0 | 0 | 17 | 0 | 1 | 364 | 1 | 1 | 1 | 1 |  |
| 80 | 1 | 0 | 0 | 17 | 0 | 1 | 364 | 1 | 1 | 1 | 1 |  |
| 81 | 1 | 0 | 0 | 17 | 0 | 1 | 364 | 1 | 1 | 1 | 1 |  |
| 82 | 1 | 0 | 0 | 17 | 0 | 1 | 364 | 1 | 1 | 1 | 1 |  |
| 83 | 1 | 0 | 0 | 17 | 0 | 1 | 364 | 1 | 1 | 1 | 1 |  |
| 84 | 1 | 0 | 0 | 17 | 0 | 2 | 364 | 1 | 1 | 1 | 1 |  |
| 85 | 1 | 0 | 0 | 17 | 0 | 1 | 364 | 1 | 1 | 1 | 1 |  |
| 86 | 1 | 0 | 0 | 17 | 0 | 1 | 364 | 1 | 1 | 1 | 1 |  |
| 87 | 1 | 0 | 0 | 17 | 0 | 1 | 364 | 1 | 1 | 1 | 1 |  |
| 88 | 1 | 0 | 0 | 17 | 0 | 1 | 364 | 1 | 1 | 1 | 1 |  |
| 89 | 1 | 0 | 0 | 17 | 0 | 1 | 364 | 1 | 1 | 1 | 1 |  |
| 90 | 1 | 0 | 0 | 17 | 0 | 1 | 364 | 1 | 1 | 1 | 1 |  |
| 91 | 1 | 0 | 0 | 17 | 0 | 1 | 364 | 1 | 1 | 1 | 1 |  |
| 92 | 1 | 0 | 0 | 17 | 0 | 1 | 364 | 1 | 1 | 1 | 1 |  |
| 93 | 1 | 0 | 0 | 17 | 0 | 1 | 364 | 1 | 1 | 1 | 1 |  |
| 94 | 1 | 0 | 0 | 17 | 0 | 1 | 364 | 1 | 1 | 1 | 1 |  |
| 95 | 1 | 0 | 0 | 17 | 0 | 1 | 364 | 1 | 1 | 1 | 1 |  |
| 96 | 1 | 0 | 0 | 17 | 0 | 1 | 364 | 1 | 1 | 1 | 1 |  |
| 97 | 1 | 0 | 0 | 17 | 0 | 1 | 364 | 1 | 1 | 1 | 1 |  |
| 98 | 1 | 0 | 0 | 17 | 0 | 1 | 364 | 1 | 1 | 1 | 1 |  |
| 99 | 1 | 0 | 0 | 17 | 0 | 1 | 364 | 1 | 1 | 1 | 1 |  |
| 100 | 1 | 0 | 0 | 17 | 0 | 1 | 364 | 1 | 1 | 1 | 1 |  |
| 101 | 1 | 0 | 0 | 17 | 0 | 1 | 364 | 1 | 1 | 1 | 1 |  |
| 102 | 1 | 0 | 0 | 17 | 0 | 1 | 364 | 1 | 1 | 1 | 1 |  |
| 103 | 1 | 0 | 0 | 17 | 0 | 1 | 364 | 1 | 1 | 1 | 1 |  |
| 104 | 1 | 0 | 0 | 17 | 0 | 1 | 364 | 1 | 1 | 1 | 1 |  |
| 105 | 1 | 0 | 0 | 17 | 0 | 1 | 364 | 1 | 1 | 1 | 1 |  |
| 106 | 1 | 0 | 0 | 17 | 0 | 1 | 364 | 1 | 1 | 1 | 1 |  |
| 107 | 1 | 0 | 0 | 17 | 0 | 1 | 364 | 1 | 1 | 1 | 1 |  |
| 108 | 1 | 0 | 0 | 17 | 0 | 2 | 364 | 1 | 1 | 1 | 1 |  |
| 109 | 1 | 0 | 0 | 17 | 0 | 2 | 364 | 1 | 1 | 1 | 1 |  |
| 110 | 1 | 0 | 0 | 17 | 0 | 1 | 364 | 1 | 1 | 1 | 1 |  |
| 111 | 1 | 0 | 0 | 17 | 0 | 1 | 364 | 1 | 1 | 1 | 1 |  |
| 112 | 1 | 0 | 0 | 17 | 0 | 1 | 364 | 1 | 1 | 1 | 1 |  |
| 113 | 1 | 0 | 0 | 17 | 0 | 1 | 364 | 1 | 1 | 1 | 1 |  |
| 114 | 1 | 0 | 0 | 17 | 0 | 1 | 364 | 1 | 1 | 1 | 1 |  |
| 115 | 1 | 0 | 0 | 17 | 0 | 2 | 364 | 1 | 1 | 1 | 1 |  |
| 116 | 1 | 0 | 0 | 17 | 0 | 2 | 364 | 1 | 1 | 1 | 1 |  |
| 117 | 1 | 0 | 0 | 17 | 0 | 1 | 364 | 1 | 1 | 1 | 1 |  |
| 118 | 1 | 0 | 0 | 17 | 0 | 1 | 364 | 1 | 1 | 1 | 1 |  |
| 119 | 1 | 0 | 0 | 17 | 0 | 1 | 364 | 1 | 1 | 1 | 1 |  |
| 120 | 1 | 0 | 0 | 17 | 0 | 2 | 364 | 1 | 1 | 1 | 1 |  |
| 121 | 1 | 0 | 0 | 17 | 0 | 1 | 364 | 1 | 1 | 1 | 1 |  |
| 122 | 1 | 0 | 0 | 17 | 0 | 1 | 364 | 1 | 1 | 1 | 1 |  |
| 123 | 1 | 0 | 0 | 17 | 0 | 2 | 364 | 1 | 1 | 1 | 1 |  |
| 124 | 1 | 0 | 0 | 17 | 0 | 1 | 364 | 1 | 1 | 1 | 1 |  |
| 125 | 1 | 0 | 0 | 17 | 0 | 1 | 364 | 1 | 1 | 1 | 1 |  |
| 126 | 1 | 0 | 0 | 17 | 0 | 1 | 364 | 1 | 1 | 1 | 1 |  |
| 127 | 1 | 0 | 0 | 18 | 1 | 1 | 364 | 1 | 1 | 1 | 1 |  |
| 128 | 1 | 0 | 0 | 18 | 1 | 1 | 364 | 1 | 1 | 1 | 1 |  |
| 129 | 1 | 0 | 0 | 18 | 1 | 1 | 364 | 1 | 1 | 1 | 1 |  |
| 130 | 1 | 0 | 0 | 18 | 1 | 1 | 364 | 1 | 1 | 1 | 1 |  |
| 131 | 1 | 0 | 0 | 18 | 1 | 1 | 364 | 1 | 1 | 1 | 1 |  |
| 132 | 1 | 0 | 0 | 18 | 1 | 1 | 364 | 1 | 1 | 1 | 1 |  |
| 133 | 1 | 0 | 0 | 18 | 1 | 2 | 364 | 1 | 1 | 1 | 1 |  |
| 134 | 1 | 0 | 0 | 18 | 1 | 1 | 364 | 1 | 1 | 1 | 1 |  |
| 135 | 1 | 0 | 0 | 18 | 1 | 1 | 364 | 1 | 1 | 1 | 1 |  |
| 136 | 1 | 0 | 0 | 18 | 1 | 1 | 364 | 1 | 1 | 1 | 1 |  |
| 137 | 1 | 0 | 0 | 18 | 1 | 1 | 364 | 1 | 1 | 1 | 1 |  |
| 138 | 1 | 0 | 0 | 18 | 1 | 1 | 364 | 1 | 1 | 1 | 1 |  |
| 139 | 1 | 0 | 0 | 18 | 1 | 1 | 364 | 1 | 1 | 1 | 1 |  |
| 140 | 1 | 0 | 0 | 18 | 1 | 1 | 364 | 1 | 1 | 1 | 1 |  |
| 141 | 1 | 0 | 0 | 18 | 1 | 1 | 364 | 1 | 1 | 1 | 1 |  |
| 142 | 1 | 0 | 0 | 18 | 1 | 1 | 364 | 1 | 1 | 1 | 1 |  |
| 143 | 1 | 0 | 0 | 18 | 1 | 2 | 364 | 1 | 1 | 1 | 1 |  |
| 144 | 1 | 0 | 0 | 18 | 1 | 1 | 364 | 1 | 1 | 1 | 1 |  |
| 145 | 1 | 0 | 0 | 18 | 1 | 1 | 364 | 1 | 1 | 1 | 1 |  |
| 146 | 1 | 0 | 0 | 18 | 1 | 1 | 364 | 1 | 1 | 1 | 1 |  |
| 147 | 1 | 0 | 0 | 18 | 1 | 1 | 364 | 1 | 1 | 1 | 1 |  |
| 148 | 1 | 0 | 0 | 18 | 1 | 1 | 364 | 1 | 1 | 1 | 1 |  |
| 149 | 1 | 0 | 0 | 18 | 1 | 1 | 364 | 1 | 1 | 1 | 1 |  |
| 150 | 1 | 0 | 0 | 18 | 1 | 1 | 364 | 1 | 1 | 1 | 1 |  |
| 151 | 1 | 0 | 0 | 18 | 1 | 1 | 364 | 1 | 1 | 1 | 1 |  |
| 152 | 1 | 0 | 0 | 18 | 1 | 1 | 364 | 1 | 1 | 1 | 1 |  |
| 153 | 1 | 0 | 0 | 18 | 1 | 1 | 364 | 1 | 1 | 1 | 1 |  |
| 154 | 1 | 0 | 0 | 18 | 1 | 1 | 364 | 1 | 1 | 1 | 1 |  |
| 155 | 1 | 0 | 0 | 18 | 1 | 1 | 364 | 1 | 1 | 1 | 1 |  |
| 156 | 1 | 0 | 0 | 18 | 1 | 2 | 364 | 1 | 1 | 1 | 1 |  |
| 157 | 1 | 0 | 0 | 18 | 1 | 1 | 364 | 1 | 1 | 1 | 1 |  |
| 158 | 1 | 0 | 0 | 18 | 1 | 1 | 364 | 1 | 1 | 1 | 1 |  |
| 159 | 1 | 0 | 0 | 18 | 1 | 1 | 364 | 1 | 1 | 1 | 1 |  |
| 160 | 1 | 0 | 0 | 18 | 1 | 1 | 364 | 1 | 1 | 1 | 1 |  |
| 161 | 1 | 0 | 0 | 18 | 1 | 1 | 364 | 1 | 1 | 1 | 1 |  |
| 162 | 1 | 0 | 0 | 18 | 1 | 1 | 364 | 1 | 1 | 1 | 1 |  |
| 163 | 1 | 0 | 0 | 18 | 1 | 1 | 364 | 1 | 1 | 1 | 1 |  |
| 164 | 1 | 0 | 0 | 18 | 1 | 1 | 364 | 1 | 1 | 1 | 1 |  |
| 165 | 1 | 0 | 0 | 18 | 1 | 1 | 364 | 1 | 1 | 1 | 1 |  |
| 166 | 1 | 0 | 0 | 18 | 1 | 1 | 364 | 1 | 1 | 1 | 1 |  |
| 167 | 1 | 0 | 0 | 18 | 1 | 1 | 364 | 1 | 1 | 1 | 1 |  |
| 168 | 1 | 0 | 0 | 19 | 0 | 1 | 364 | 1 | 1 | 1 | 1 |  |
| 169 | 1 | 0 | 0 | 19 | 0 | 1 | 364 | 1 | 1 | 1 | 1 |  |
| 170 | 1 | 0 | 0 | 19 | 0 | 1 | 364 | 1 | 1 | 1 | 1 |  |
| 171 | 1 | 0 | 0 | 19 | 0 | 1 | 364 | 1 | 1 | 1 | 1 |  |
| 172 | 1 | 0 | 0 | 19 | 0 | 1 | 364 | 1 | 1 | 1 | 1 |  |
| 173 | 1 | 0 | 0 | 19 | 0 | 1 | 364 | 1 | 1 | 1 | 1 |  |
| 174 | 1 | 0 | 0 | 19 | 0 | 1 | 364 | 1 | 1 | 1 | 1 |  |
| 175 | 1 | 0 | 0 | 19 | 0 | 2 | 364 | 1 | 1 | 1 | 1 |  |
| 176 | 1 | 0 | 0 | 19 | 0 | 2 | 364 | 1 | 1 | 1 | 1 |  |
|  | 1 | 0 | 0 | 19 | 0 | 2 | 364 | 1 | 1 | 1 | 1 |  |
| 178 | 1 | 0 | 0 | 19 | 0 | 2 | 364 | 1 | 1 | 1 | 1 |  |
| 179 | 1 | 0 | 0 | 19 | 0 | 1 | 364 | 1 | 1 | 1 | 1 |  |
| 180 | 1 | 0 | 0 | 19 | 0 | 1 | 364 | 1 | 1 | 1 | 1 |  |
| 181 | 1 | 0 | 0 | 19 | 0 | 1 | 364 | 1 | 1 | 1 | 1 |  |
| 182 | 1 | 0 | 0 | 19 | 0 | 1 | 364 | 1 | 1 | 1 | 1 |  |
| 183 | 1 | 0 | 0 | 19 | 0 | 1 | 364 | 1 | 1 | 1 | 1 |  |
| 184 | 1 | 0 | 0 | 19 | 0 | 1 | 364 | 1 | 1 | 1 | 1 |  |
| 185 | 1 | 0 | 0 | 19 | 0 | 1 | 364 | 1 | 1 | 1 | 1 |  |
| 186 | 1 | 0 | 0 | 19 | 0 | 2 | 364 | 1 | 1 | 1 | 1 |  |
| 187 | 1 | 0 | 0 | 19 | 0 | 1 | 364 | 1 | 1 | 1 | 1 |  |
| 188 | 1 | 0 | 0 | 19 | 0 | 1 | 364 | 1 | 1 | 1 | 1 |  |
| 189 | 1 | 0 | 0 | 19 | 0 | 1 | 364 | 1 | 1 | 1 | 1 |  |
| 190 | 1 | 0 | 0 | 19 | 0 | 1 | 364 | 1 | 1 | 1 | 1 |  |
| 191 | 1 | 0 | 0 | 19 | 0 | 1 | 364 | 1 | 1 | 1 | 1 |  |
| 192 | 1 | 0 | 0 | 19 | 0 | 1 | 364 | 1 | 1 | 1 | 1 |  |
| 193 | 1 | 0 | 0 | 19 | 0 | 1 | 364 | 1 | 1 | 1 | 1 |  |
| 194 | 1 | 0 | 0 | 19 | 0 | 1 | 364 | 1 | 1 | 1 | 1 |  |
| 195 | 1 | 0 | 0 | 19 | 0 | 1 | 364 | 1 | 1 | 1 | 1 |  |
| 196 | 1 | 0 | 0 | 19 | 0 | 1 | 364 | 1 | 1 | 1 | 1 |  |
| 197 | 1 | 0 | 0 | 19 | 0 | 1 | 364 | 1 | 1 | 1 | 1 |  |
| 198 | 1 | 0 | 0 | 19 | 0 | 2 | 364 | 1 | 1 | 1 | 1 |  |
| 199 | 1 | 0 | 0 | 19 | 0 | 1 | 364 | 1 | 1 | 1 | 1 |  |
| 200 | 1 | 0 | 0 | 19 | 0 | 1 | 364 | 1 | 1 | 1 | 1 |  |
| 201 | 1 | 0 | 0 | 19 | 0 | 1 | 364 | 1 | 1 | 1 | 1 |  |
| 202 | 1 | 0 | 0 | 19 | 0 | 1 | 364 | 1 | 1 | 1 | 1 |  |
| 203 | 1 | 0 | 0 | 19 | 0 | 1 | 364 | 1 | 1 | 1 | 1 |  |
| 204 | 1 | 0 | 0 | 19 | 0 | 2 | 364 | 1 | 1 | 1 | 1 |  |
| 206 | 1 |  | 1 | 18 | 1 | 1 | 364 |  |  |  |  |  |
| 207 | 1 |  | 1 | 18 | 1 | 1 | 364 |  |  |  |  |  |
| 208 | 1 |  | 1 | 18 | 1 | 1 | 364 |  |  |  |  |  |
| 209 | 1 |  | 1 | 18 | 1 | 1 | 364 |  |  |  |  |  |
| 231 | 1 |  | 1 | 15 | 1 | 1 | 364 |  |  |  |  |  |
| 232 | 1 |  | 1 | 15 | 1 | 1 | 364 |  |  |  |  |  |
| 233 | 1 |  | 1 | 18 | 1 | 1 | 364 |  |  |  |  |  |
| 234 | 1 |  | 1 | 17 | 0 | 1 | 364 |  |  |  |  |  |
| 235 | 1 |  | 1 | 15 | 1 | 1 | 364 |  |  |  |  |  |
| 236 | 1 |  | 1 | 17 | 0 | 1 | 364 |  |  |  |  |  |
| 237 | 1 |  | 1 | 18 | 1 | 1 | 364 |  |  |  |  |  |
| 238 | 1 |  | 1 | 17 | 0 | 1 | 364 |  |  |  |  |  |
| 239 | 1 |  | 1 | 17 | 0 | 1 | 364 |  |  |  |  |  |

**Table S2 Information of workers with malaria test positivity**

| **Micr (（1 =Pv, 2=Pf)** | **Detect (1=active，2=passive)** | **Team number** | **Sex（1 = f， 0 =m）** | **Occupation** | **Attack site (1=project site；2=home)** | **Test site** | **Report facilities** |
| --- | --- | --- | --- | --- | --- | --- | --- |
| 1 | 1 | 18 | 1 | worker | 2 | Mangshi CDC | Mangshi CDC |
| 1 | 1 | 18 | 1 | worker | 2 | Yingjiang hospital | Yingjiang hospital |
| 1 | 2 | 18 | 1 | worker | 2 | Mangshi CDC | Mangshi CDC |
| 1 | 1 | 18 | 1 | worker | 2 | Tengchong hospitals | Tengchong hospitals |
| 1 | 1 | 15 | 1 | worker | 1 | Yingjiang hospital | Yingjiang hospital |
| 1 | 1 | 15 | 1 | worker | 1 | Yingjiang hospital | Yingjiang hospital |
| 1 | 1 | 18 | 1 | worker | 2 | Mangshi CDC | Mangshi CDC |
| 1 | 1 | 17 | 1 | worker | 2 | Tengchong hospitals | Tengchong hospitals |
| 1 | 2 | 15 | 1 | worker | 1 | Project site | Yingjiang hospital |
| 1 | 2 | 15 | 1 | worker | 1 | Project site | Yingjiang hospital |
| 1 | 2 | 15 | 1 | worker | 1 | Project site | Yingjiang hospital |
| 1 | 1 | 15 | 1 | worker | 1 | Xueli malaria border post | Yingjiang hospital |
| 1 | 2 | 15 | 1 | worker | 1 | Project site | Yingjiang hospital |
| 1 | 1 | 17 | 1 | worker | 1 | Project site | Yingjiang hospital |
| 1 | 2 | 15 | 0 | worker | 1 | Project site | Yingjiang CDC |
| 1 | 2 | 15 | 0 | worker | 1 | Project site | Yingjiang CDC |
| 1 | 2 | 16 | 1 | worker | 1 | Project site | Yingjiang CDC |
| 1 | 1 | 18 | 1 | worker | 2 | Tengchong hospitals | Tengchong hospitals |
| 1 | 2 | 16 | 1 | worker | 1 | Project site | Yingjiang CDC |
| 1 | 2 | 15 | 1 | worker | 1 | Project site | Yingjiang CDC |
| 1 | 1 | 17 | 1 | worker | 1 | Yingjiang hospital | Yingjiang hospital |
| 1 | 1 | 17 | 1 | worker | 1 | Yingjiang hospital | Yingjiang hospital |
